# Proximal tubule transferrin uptake is modulated by cellular iron and mediated by apical membrane megalin–cubilin complex and transferrin receptor 1

**DOI:** 10.1074/jbc.RA118.006390

**Published:** 2019-03-04

**Authors:** Craig P. Smith, Wing-Kee Lee, Matthew Haley, Søren B. Poulsen, Frank Thévenod, Robert A. Fenton

**Affiliations:** From the ‡ School of Medical Sciences, The University of Manchester, Manchester M13 9PT, United Kingdom,; § Physiology, Pathophysiology and Toxicology, University of Witten/Herdecke, D-58453 Witten, Germany, and; ¶ Department of Biomedicine, Aarhus University, Aarhus, 8000 Denmark

**Keywords:** transferrin, kidney, iron, membrane transport, epithelial cell, renal physiology, chelation, cubilin, endocytosis, megalin, transferrin receptor

## Abstract

Receptor-mediated endocytosis is responsible for reabsorption of transferrin (Tf) in renal proximal tubules (PTs). Although the role of the megalin–cubilin receptor complex (MCRC) in this process is unequivocal, modalities independent of this complex are evident but as yet undefined. Here, using immunostaining and Tf-flux assays, FACS analysis, and fluorescence imaging, we report localization of Tf receptor 1 (TfR1), the cognate Tf receptor mediating cellular holo-Tf (hTf) acquisition, to the apical brush border of the PT, with expression gradually declining along the PT in mouse and rat kidneys. In functional studies, hTf uptake across the apical membrane of cultured PT epithelial cell (PTEC) monolayers increased in response to decreased cellular iron after desferrioxamine (DFO) treatment. We also found that apical hTf uptake under basal conditions is receptor-associated protein (RAP)-sensitive and therefore mediated by the MCRC but becomes RAP-insensitive under DFO treatment, with concomitantly decreased megalin and cubilin expression levels and increased TfR1 expression. Thus, as well as the MCRC, TfR1 mediates hTf uptake across the PT apical brush border, but in conditions of decreased cellular iron, hTf uptake is predominated by augmented apical TfR1. In conclusion, both the MCRC and TfR1 mediate hTf uptake across apical brush border membranes of PTECs and reciprocally respond to decreased cellular iron. Our findings have implications for renal health, whole-body iron homeostasis, and pathologies arising from disrupted iron balance.

## Introduction

The renal proximal tubule (PT)^2^ is responsible for bulk reabsorption of a spectrum of essential solutes filtered by the glomeruli. PT dysfunction is a characteristic of several disorders, including acute renal injury, chronic kidney disease, and cadmium toxicity (1). Recently, iron handling by the kidney has attracted considerable interest. This is triggered by the localization of the proton-coupled iron transporter protein divalent metal transporter 1 (SLC11A2; DMT1) to the kidney (2) plus the discovery of iron in the primary glomerular filtrate and unidirectional iron flux out of the PT (3–5).

Particularly striking are the considerable amounts of DMT1 in kidney PT epithelial cells (PTECs) (6) and the finding that levels of DMT1 and the iron export protein ferroportin 1 (SLC40A1; FPN1) expressed in PTECs are sensitive to modulation by dietary iron (7–9). This has led to the suggestion that these proteins may facilitate the renal reabsorption of urinary iron and in so doing contribute to homeostatic control of body iron balance (4). In mouse and rat PTs, DMT1 has been localized to intracellular vesicles corresponding to late endosomes and lysosomes (10). In contrast, FPN1 localizes to basolateral membranes in rat and human PTs (9, 11) and could possibility mediate flux of iron from PTECs into the blood (9).

Although the components to enable transepithelial flux of iron are resident in PTECs and several research groups have studied their function, the mechanism(s) by which iron is reabsorbed from the primary filtrate remains controversial. This issue is compounded by the fact that it is still not established in what form iron, protein-bound, free, or both, is transported into PTECs (12, 13). Several candidate receptors and solute transporters that mediate transport of protein-bound iron or free iron have been proposed, including the multiligand megalin–cubilin receptor complex (MCRC (14)), the transferrin receptor 1 (TfR1 (15)), DMT1 (16), zinc transporter Zip 8 (17), and zinc transporter Zip 14 (Ref. 17; for a review, see Ref. 5). For the majority, with the exception of MCRC, clear functional evidence is lacking (13).

Because in the absence of disease there is a negligible amount of nonprotein-bound iron in plasma (18) and given that a fraction of the ubiquitous iron-binding plasma protein transferrin (Tf) is filtered by the glomerulus and reabsorbed along the PT (19, 20), it is likely that iron bound to Tf may be one form of iron that is transported into PTECs. Supporting this notion, the MCRC is expressed in the apical membrane of PTECs and mediates endocytosis of a spectrum of ligands, including Tf (13, 21). Tf is also detected in urine from mice deficient in MCRC, thus implicating this receptor complex in the reabsorption of filtered Tf (22, 23). Tf is the cognate ligand for the ubiquitously expressed TfR1. Cells acquire essential iron by TfR1-mediated uptake of holotransferrin (hTf). There is, however, scant data detailing the renal expression of TfR1, but the existing published data point to TfR1 expression in PTECs, and accordingly, TfR1 has been suggested to affect apical uptake of filtered Tf (15). Functional data supporting this suggestion are lacking.

The aim of the current study was to determine the precise location of TfR1 in the proximal tubule and to assess the mechanistic significance of TfR1 expression in PTECs by measuring hTf uptake across the apical or basolateral membrane of polarized cultured PTECs. The mode of hTf uptake was assessed by performing ligand competition studies in iron-replete PTECs and PTECs with low cellular iron. We determine that TfR1 is expressed on the apical cell surface of PTECs *in vivo* and in cultured human and rat PT cells. hTf internalization across the apical membrane of PTECs is mediated by both MCRC and TfR1. The predominant receptor mediating uptake depends on cellular iron content. These results have major implications for renal health, whole-body iron homeostasis, and pathologies where iron balance is disrupted.

## Results

### TfR1 is expressed in the proximal tubule apical membrane

**Figure 1.**
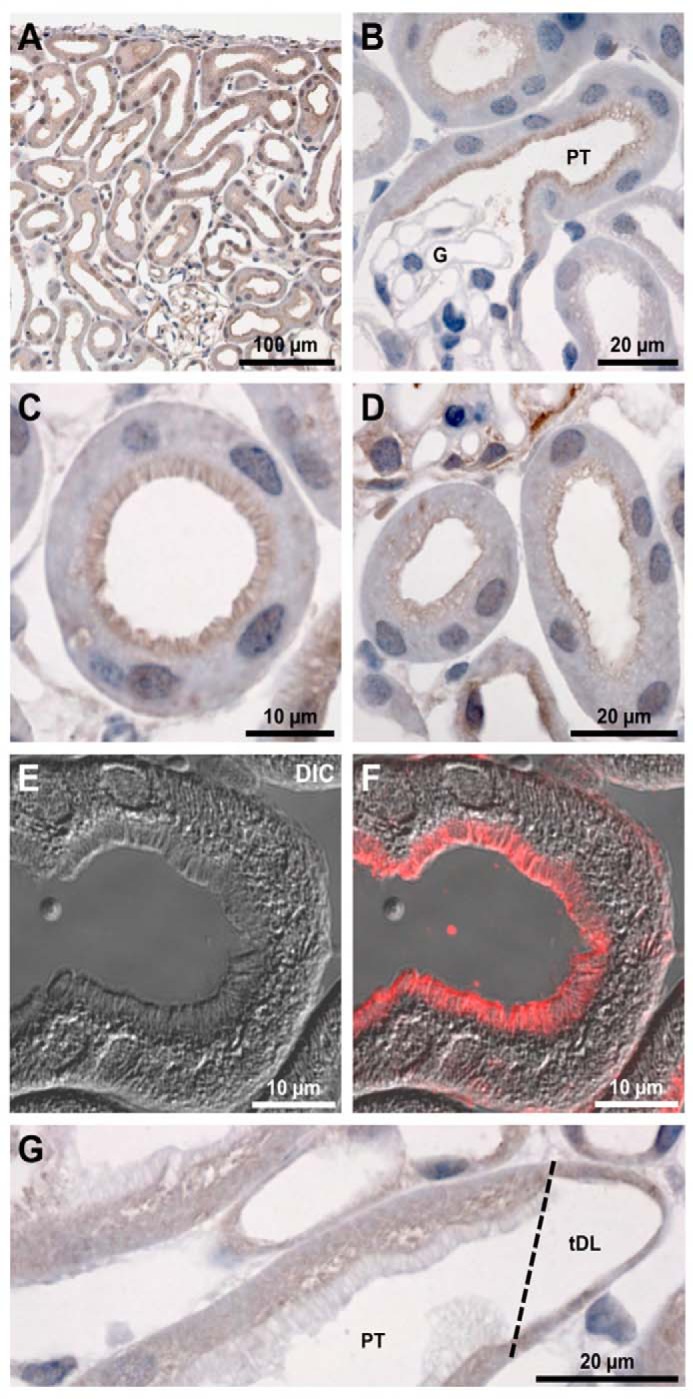
**Immunohistochemistry of TfR1 in mouse kidney sections.** *A*, staining is apparent throughout the cortex, predominantly at the luminal membrane domain of PTs. *B*, at higher magnification, clear luminal staining is apparent at the apical surface of early PTs, with no staining of the glomerulus (*G*). *C* and *D*, staining was strongest in the brush border of early PTs and was weaker in late PT. *E* and *F*, confocal microscopy coupled to differential interference contrast (*DIC*) imaging highlights the labeling of the brush border of PT cells. *G*, TfR was absent from the very late PT where it transitions to the descending thin limb of Henle's loop (*tDL*).

In mouse kidney, TfR1 was detected in the proximal tubules originating from both superficial and deep-lying nephrons, with the strongest labeling detected in the apical brush border of PTECs (Fig. 1, *A* and *B*). Confocal laser-scanning microscopy coupled to differential interference contrast imaging confirmed the predominant apical localization of TfR1 in PTECs (Fig. 1, *E* and *F*). Apically associated TfR1 immunostaining was strongest in the early proximal tubule (Fig. 1*C*), gradually decreased along the length of the tubule (Fig. 1*D*), and was essentially absent in the brush border of tubules transitioning to thin descending limbs of Henle's loop (Fig. 1*G*). A similar localization of TfR1 was observed in rat kidney (not shown).

### Polarized uptake of transferrin into Wistar–Kyoto rat proximal tubule (WKPT) cells

**Figure 2.**
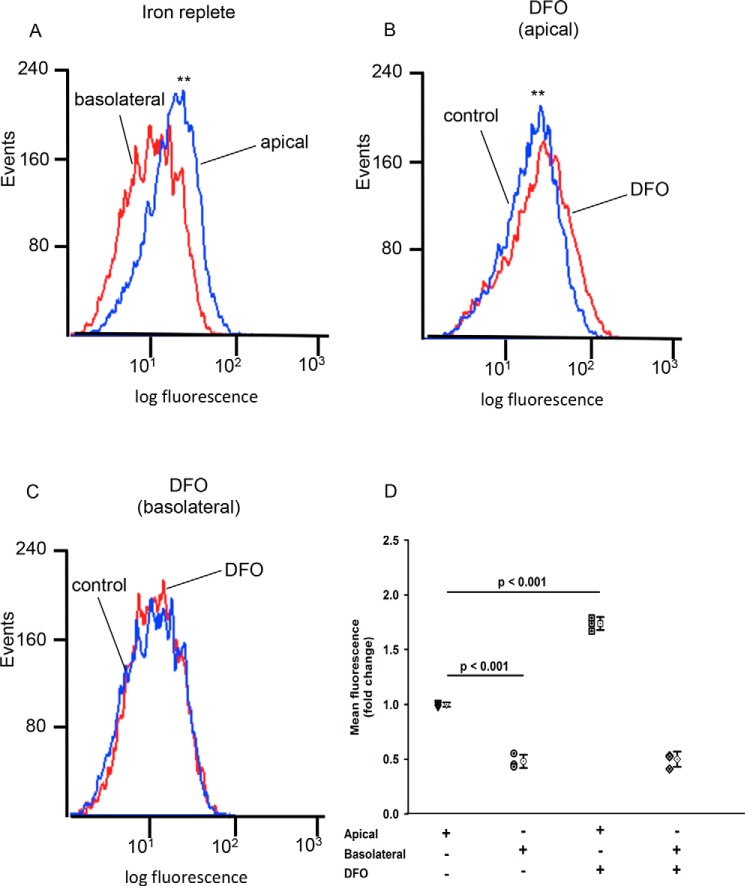
**Iron chelation induces apical membrane uptake of Alexa 488 holotransferrin.** Shown is representative flow cytometric (FACS) analysis of Alexa 488-hTf uptake into polarized WKPT cells grown on permeable supports. *A*, under iron-replete control conditions, mean uptake of Alexa 488-hTf from the apical compartment (*blue*) was significantly greater than mean uptake from the basolateral compartment (*red*) (**, *p* < 0.001 control apical *versus* control basolateral). *B*, following treatment with DFO, mean uptake of Alexa 488-hTf from the apical compartment was significantly greater than apical uptake under control conditions (*blue*) (**, *p* < 0.001 control apical *versus* DFO apical). *C*, low cellular iron instilled by DFO treatment did not affect cellular uptake of Alexa 488-hTf from the basolateral compartment. Kolmogorov–Smirnov statistical analysis was used to analyze cumulative frequency. *D*, summary of mean Alexa 488-hTf fluorescence uptake into WKPT-0293 Cl.2. Means represent *n* = 3. *Error bars* represent S.D. ±S.D., 10,000 events per replicate.

To determine the effect of decreased cellular iron on hTf uptake into polarized WKPT cells, we performed experiments whereby Alexa Fluor 488-holotransferrin (A488-hTf) was added to either the apical or basolateral chambers of Transwells containing confluent WKPT cells and then measured cellular fluorescence using FACS (Fig. 2). Uptake of hTf from the apical compartment was 2-fold greater than from the basolateral compartment under iron-replete conditions (Fig. 2, *A* and *D*; *p* ≤ 0.001, *n* = 3). Pretreatment of cells with desferrioxamine (DFO) to reduce cellular iron content resulted in a ∼50% increase in mean cellular fluorescence when Alexa 488-hTf was added to the apical compartment (Figs. 2, *B* and *D*; *p* < 0.001, *n* = 3), but mean fluorescence did not significantly change compared with the iron-replete state when A488-hTf was added to the basolateral compartment (Fig. 2*C*). Pulse-chase experiments with an hTf pulse for 30 min followed by a 45-min chase clearly showed that reduced cellular iron caused a marked increase in hTf uptake that was sustained throughout the chase time course (Fig. S2). These data indicate that hTf uptake across the apical membrane was greater than hTf uptake across the basolateral membrane and that uptake across the apical membrane, but not the basolateral membrane, was increased in response to a reduction in cellular iron levels.

**Figure 3.**
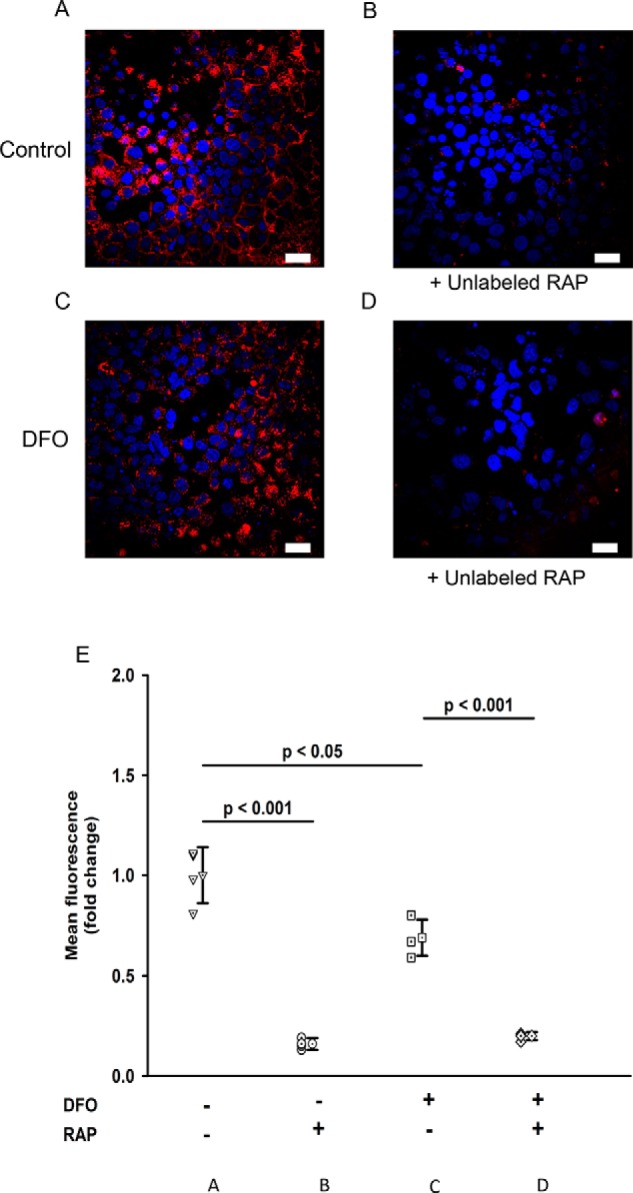
**Uptake of RAP by WKPT cells.** *A–D* are representative micrographs of WKPT cells grown on coverslips and treated as follows. *A*, incubation of WKPT cells with Cy3-RAP resulted in an increase in cellular fluorescence indicating Cy3-RAP uptake. *B*, inclusion of a 50× molar excess of unlabeled RAP caused a significant decrease in Cy3-RAP compared with control cells. *C*, pretreatment of cells with DFO to lower cellular iron caused a decrease in cellular Cy3-RAP fluorescence. *D*, incubation of DFO-pretreated WKPT cells with Cy3-RAP and a 50× molar excess of unlabeled RAP caused a significant decrease in cellular fluorescence compared with DFO-treated control cell. *E*, scatter graph summary of mean Cy3-RAP uptake by WKPT cells. Means represents *n* = 4 replicates. *Error bars* represent S.D. ±S.D. Levels of significance were determined by ANOVA and Dunnett's post hoc test. *Scale bars*, 20 μm.

We then sought to determine the mechanism responsible for hTf uptake in iron-replete cells and compared this with cells with reduced cellular iron. To do this, cells were grown on coverslips, and uptake of fluorescent proteins across the apical membrane was measured. We first performed experiments to probe the role of the MCRC using RAP. Under control iron-replete conditions, cells avidly took up Cy3-RAP (Fig. 3*A*), and Cy3-RAP uptake was 75% reduced by addition of a 50-fold excess of unlabeled RAP (Fig. 3, *B* and *E*; *p* < 0.001, *n* = 4). This finding confirmed that MCRC was functional in WKPT cells and that treatment with RAP was a viable means of inhibiting Cy3-RAP uptake via MCRC. We then examined the effect of reduced cellular iron on Cy3-RAP uptake. Cells rendered low in iron by exposure to DFO took up significantly less Cy3-RAP compared with control iron-replete cells (Fig. 3, *C* and *E*; *p* < 0.05, *n* = 4). As observed for control cells, excess unlabeled RAP significantly reduced Cy3-RAP uptake to a level that was similar to that recorded for control cells (Fig. 3, *D* and *E*; *p* < 0.001, *n* = 4). These results suggest that DFO treatment reduces MCRC-mediated RAP uptake.

**Figure 4.**
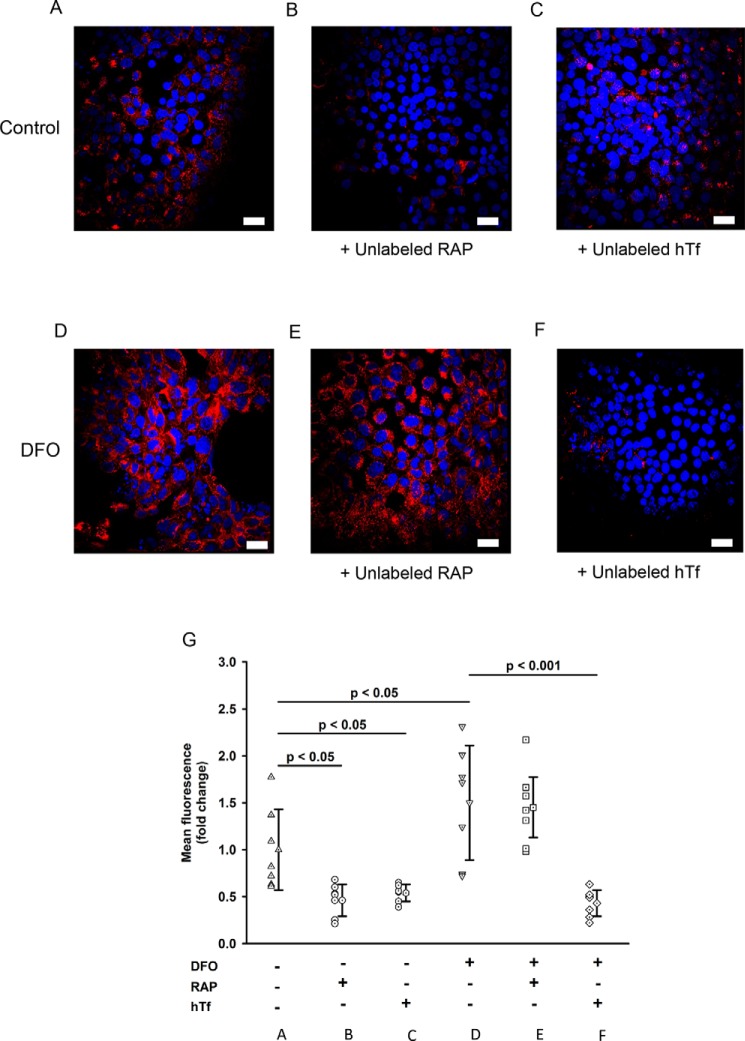
**Uptake of Alexa 488-holotransferrin (hTf) by WKPT cells.** *A–F* are representative micrographs of WKPT cells grown on coverslips and treated as follows. *A*, incubation of WKPT cells with Alexa 488-hTf resulted in an increase in cellular fluorescence. *B*, inclusion of a 50× molar excess of unlabeled RAP caused a significant decrease in Alexa 488-hTf compared with control cells (*p* < 0.05). *C*, incubation of WKPT cells with Alexa 488-hTf and a 50× molar excess of unlabeled hTf caused a significant decrease in cellular fluorescence compared with control cells (*p* < 0.05). *D*, pretreatment of cells with DFO to lower cellular iron caused an increase in cellular Alexa 488-hTf fluorescence compared with control cells (*p* < 0.05). *E*, incubation of DFO-pretreated WKPT cells with Alexa 488-hTf and a 50× molar excess of unlabeled RAP had no significant effect on cellular fluorescence compared with DFO-treated control cells. *F*, a 50× molar excess of hTf decreased uptake of Alexa 488-hTf into DFO-pretreated WKPT cells compared with DFO-pretreated control cells (*p* < 0.001). *Scale bars*, 20 μm. *G*, scatter graph summary of mean Alexa 488-hTf uptake into WKPT-0293 Cl.2. Control cells were incubated with either Alexa 488-hTf, Alexa 488-hTf + 50× unlabeled RAP, or Alexa 488-hTf + 50× unlabeled hTf. Cells were also pretreated with DFO to lower cellular iron and then incubated with Alexa 488-hTf, Alexa 488-hTf + 50× unlabeled RAP, or Alexa 488-hTf + 50× unlabeled hTf. *Error bars* represent S.D.

Next, we performed experiments to determine whether hTf uptake into WKPT cells was RAP-sensitive. Incubation of control cells with Alexa 488-hTf resulted in an increase in cellular fluorescence, indicating that labeled hTf was internalized by the cells (Fig. 4). Inclusion of a 50-fold molar excess of RAP significantly reduced Alexa 488-hTf uptake by 50% (Fig. 4, *B* and *G*; *p* < 0.05, *n* = 7), indicating that hTf uptake was mediated by a RAP-sensitive pathway. Reducing cellular iron by exposing cells to DFO caused Alexa 488-hTf uptake to increase by 50% (Fig. 4, *D* and *G*; *p* < 0.05, *n* = 7). Importantly, under these conditions, inclusion of a 50-fold molar excess of RAP did not significantly reduce Alexa 488-hTf uptake (Fig. 4, *E* and *G*). We then repeated the experiment except in the presence of unlabeled hTf and observed a decrease in Alexa 488-hTf uptake in both control and DFO groups compared with iron-replete control cells (*p* < 0.05 and *p* < 0.001, respectively, *n* = 7). This indicates that in iron-depleted cells A488-hTf uptake is reduced by hTf but not RAP. Taken together, under iron-replete conditions, hTf uptake was found to be sensitive to RAP and therefore was mediated in part by the MCRC; however, when cellular iron was reduced, hTf uptake by MCRC decreased but remained hTf-sensitive.

### Quantitative RT-PCR (RT-qPCR)

**Figure 5.**
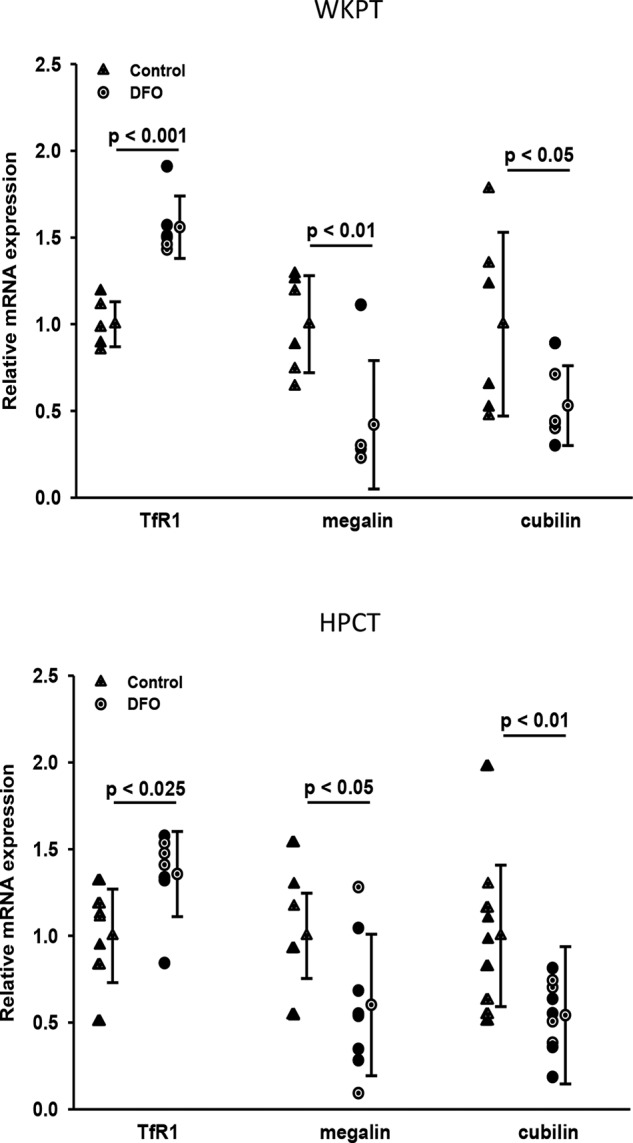
**RT-qPCR analysis of TfR1, megalin, and cubilin cDNA from proximal tubule cells following DFO treatment.** Cells were treated for 24 h with 100 μm DFO. Quantitative real-time PCR products encoding TfR1, megalin, and cubilin were amplified using cDNA reverse transcribed from total RNA isolated from WKPT (*A*) or HPCT (*B*). DFO treatment (*circles*) led to an increase in TfR1 mRNA, whereas megalin and cubilin cDNAs decreased compared with untreated controls. Values represent the means from six to nine independent experiments. *Error bars* represent S.D. Significance relates to DFO treatment group *versus* corresponding control group.

RT-qPCR was utilized to quantify mRNA transcripts encoding TfR1, megalin, and cubilin. Transcripts encoding TfR1 were significantly increased following 24-h exposure of WKPT or human proximal tubule (HPCT) cells to DFO (*p* < 0.001 and *p* < 0.025, respectively, *n* = 6; Fig. 5). In contrast, mRNA encoding megalin or cubilin was significantly decreased in both cell lines (*p* < 0.05–0.01, *n* = 6–9; Fig. 5). These findings corroborate and reinforce our functional studies showing decreased RAP-sensitive hTf uptake and an increase in RAP-insensitive hTf uptake.

### Immunofluorescence staining of TfR1 and megalin

**Figure 6.**
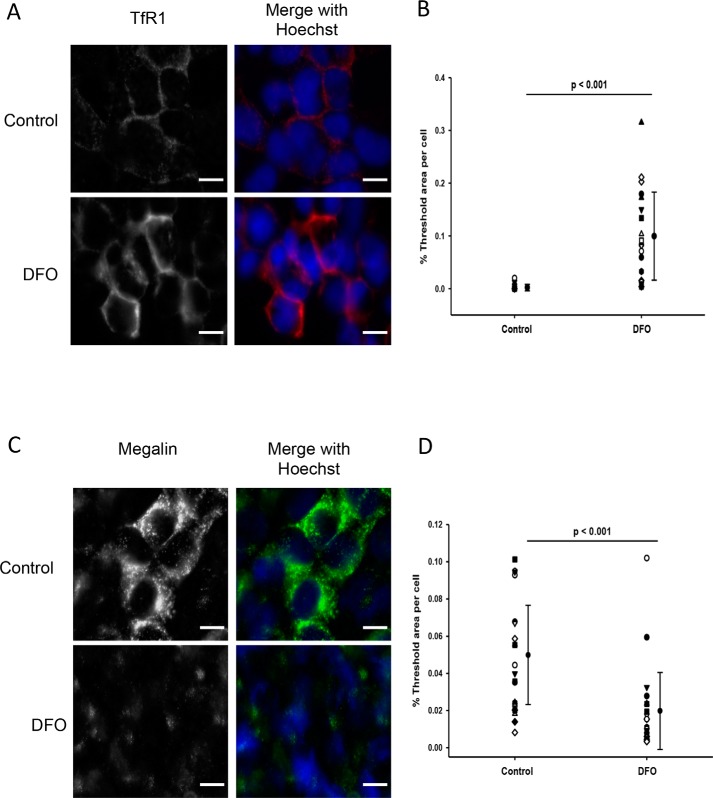
**Immunofluorescence staining of TfR1 (*A* and *B*) and megalin (*C* and *D*) in WKPT cells following DFO treatment.** Confluent WKPT-0293 cells were treated with 0.1 mm DFO for 24 h, fixed, and immunostained for TfR1 using a PE-conjugated antibody at 1:200 or for megalin (1:125) in combination with A488-coupled secondary antibody (1:500). Nuclei were stained with Hoechst. Images are representative of three independent experiments. *A*, TfR1 receptor expression increased in low-iron cells. *B*, summary of densitometric analysis of TfR1 signal. Means are shown. *Error bars* represent S.D. (*n* = 15–20 images). Student's unpaired *t* test compares DFO with serum-free medium. *Scale bars*, 10 μm. *C*, megalin expression decreased in low-iron cells. *D*, summary of densitometric analysis of megalin signal. Means ± S.D. (*n* = 18 images) are shown. Student's unpaired *t* test compares DFO with control. *Scale bars*, 10 μm.

**Figure 7.**
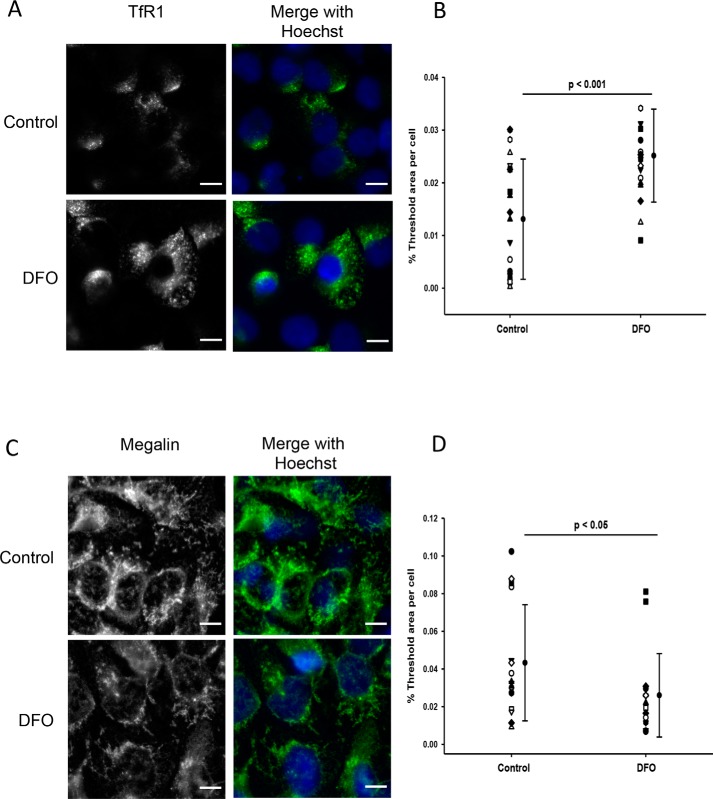
**Immunofluorescence staining of TfR1 (*A* and *B*) and megalin (*C* and *D*) in HPCT cells following DFO treatment.** Confluent HPCT cells were treated with 0.1 mm DFO for 24 h, fixed, and immunostained for TfR1 at 1:200 or megalin at 1:100 in combination with A488-coupled secondary antibody (1:500). *A*, TfR1 receptor expression increased in low-iron cells. *B*, summary of densitometric analysis of TfR1 signal. Means are shown. *Error bars* represent S.D. (*n* = 18 images). Student's unpaired *t* test compares DFO with control. *Scale bars*, 10 μm. *C*, megalin expression decreased in low-iron cells. *D*, summary of densitometric analysis of megalin signal. Means ± S.D. (*n* = 15–16 images) are shown. Student's unpaired *t* test compares DFO with control. *Scale bars*, 10 μm.

Using immunocytochemistry, treatment of WKPT or HPCT cells with DFO for 24 h did not clearly alter TfR1 distribution but significantly increased TfR1 abundance ∼38-fold in WKPT cells (*p* < 0.001, *n* = 15–20; Fig. 6*C*) and ∼2-fold in HPCT cells (*p* < 0.001, *n* = 18; Fig. 7*C*). In contrast, similar experiments showed that DFO treatment significantly reduced megalin labeling intensity in both WKPT (*p* < 0.001, *n* = 26; Fig. 6*F*) and HPCT cells (*p* < 0.05, *n* = 15–16; Fig. 7*F*). Furthermore, megalin distribution appeared more intracellular following DFO treatment, suggesting that megalin trafficking may have been affected by DFO treatment.

### Surface biotinylation to assess plasma membrane levels of TfR1

**Figure 8.**
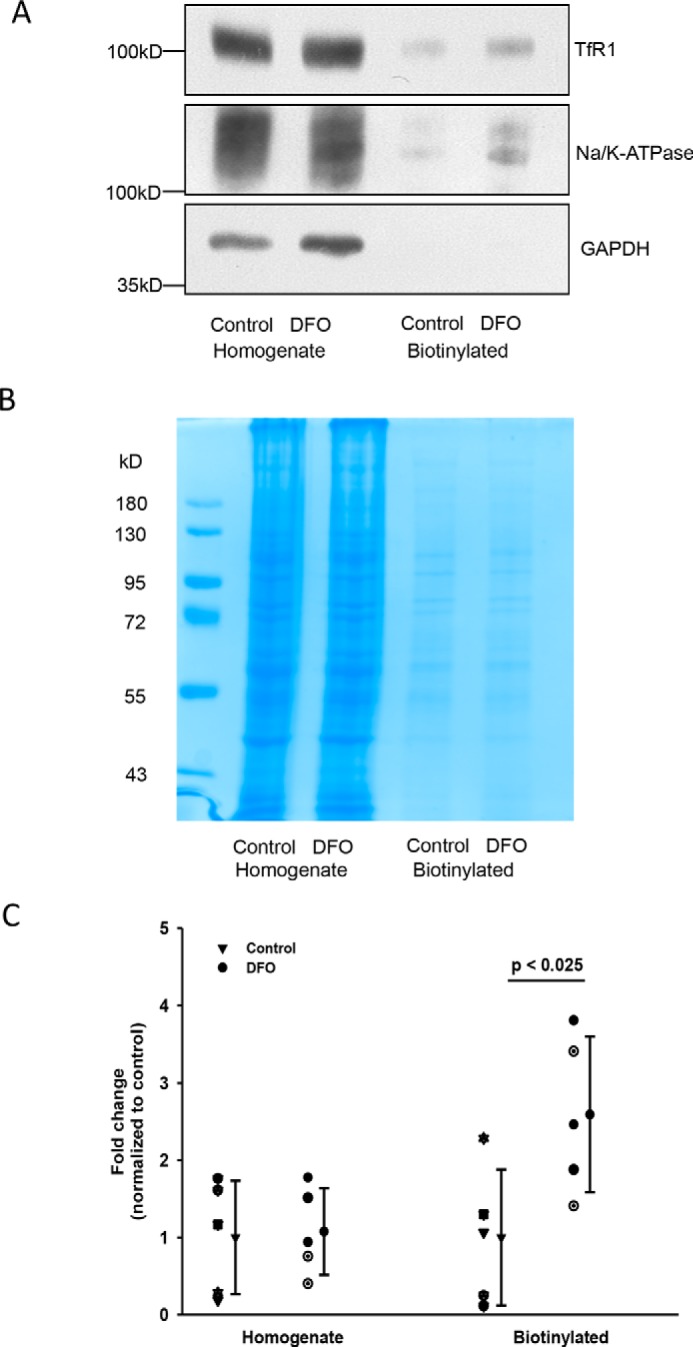
**Surface expression of Tfr1 in WKPT cells following DFO treatment.** Confluent WKPT-0293 cells grown on collagen were exposed to control medium or 0.1 mm DFO for 16 h. Apical membranes were labeled, isolated by surface biotinylation, and subjected to immunoblot analysis. *A*, representative immunoblot showing TfR1, Na/K-ATPase, and GAPDH. *B*, parallel acrylamide gels were controlled for loading using Coomassie Brilliant Blue staining. *C*, scatter plot showing densitometry analysis of TfR1 signals. Means are shown. *Error bars* represent S.D. (*n* = 5). Student's unpaired *t* test compares DFO with control.

Surface biotinylation experiments followed by densitometry analysis of biotinylated TfR1 immunoblots showed that DFO induced a 2.5-fold increase in membrane-resident TfR1 (*p* < 0.025, *n* = 5; Fig. 8*C*). Taken together, these data corroborate results from both our functional and quantitative real-time PCR (qPCR) experiments and strongly suggest that decreased cellular iron levels increase apical membrane TfR1 expression, ultimately resulting in greater hTf uptake.

## Discussion

The aims of the current study were to determine the cellular location of TfR1 in the renal PT and to extend our functional understanding of the mechanism of hTf uptake into PTECs. In addition, we also assessed whether uptake of hTf was modulated in response to lowered cellular iron content. Our interest in this mechanism stems from our general quest to understand how the renal PT transports iron.

Immunostaining of mouse and rat kidneys showed TfR1 in the apical brush border membrane of PTECs. TfR1 immunostaining was strongest in the early proximal tubule, gradually decreased along the length of the tubule, and was absent in the brush border of the regions transitioning to thin descending limbs of Henle's loop. This finding was corroborated by *in vitro* immunostaining of WKPT and HPCT cells and surface biotinylation studies that showed labeling of TfR1 on the plasma membrane of cultured human and rat PTECs. Therefore, in human, rat, and mouse, these data together give strong support to the notion that TfR1 is involved in the uptake of hTf from the primary urine. The tubular distribution of TfR1 also matches our previously described immunolocalization of DMT1 and FPN1, indicating that these proteins contribute to affect reabsorption of urinary hTf (6, 9).

Two receptor-mediated modalities with the capacity to bind and translocate hTf are indicated in the proximal tubule: the polyligand MCRC (13, 14) and the ubiquitously expressed TfR1 (25–27). The MCRC was active in our model PTECs, and under conditions when cellular iron was replete, the MCRC was partly responsible for hTf uptake. When cellular iron content was restricted, however, MCRC-mediated uptake of RAP or hTf was reduced, and this was paralleled by a reduction in both megalin and cubilin. *In vivo* studies have clearly shown a role for megalin in renal Tf uptake (22, 23), with the appearance of Tf in urine in mouse models deficient in functional megalin. Cubilin mediates Tf endocytosis in PT (13), and its role appears dependent on megalin (13). However, the MCRC alone does not fully account for PT Tf uptake because Amsellem *et al*. (22) reported cubilin-independent Tf uptake in cubilin knockout mice, pointing to an alternative mechanism for renal reuptake of Tf.

The 24p3/neutrophil gelatinase-associated lipocalin 2 receptor (Lcn2-R) has an affinity for Tf and is a potential candidate that might explain non-MCRC–mediated uptake of Tf. However, Lcn2-R is expressed in the distal tubule and inner medullary collecting duct, not in the PT, and hence is unlikely to be responsible for the non-MCRC–mediated uptake of hTf (28).

In support of a role for TfR1 in the renal reabsorption of filtered hTf is our observation that under low-iron conditions hTf uptake became insensitive to RAP but remained sensitive to hTf. In parallel, low cellular iron induced an increase in TfR1 mRNA and apical membrane TfR1 protein in well-characterized cellular models of rat and human proximal tubule cells (29, 30). Because we used ligand competition to dissect the roles of the various receptors of interest, it was not possible to ascertain completely whether jointly the MCRC and TfR1 alone were responsible for all apical uptake of hTf. Clearly, the residual uptake we observed could be due to incomplete inhibition of uptake. However, despite these limitations, our data clearly indicate a role for MCRC and TfR1 in renal hTf reabsorption, especially under low cellular iron conditions.

Previously, we have reported in the whole animal the response to iron of the two major iron transporter proteins, DMT1 and FPN1, and this led to the suggestion that these proteins were part of a mechanism to enable reabsorption of filtered iron (4). Despite previously proposed models, the nature of the apical membrane transport process responsible for iron uptake has remained obscure. The findings of the current study throw light onto this and suggest that both MCRC and TfR1 mediate uptake of iron-bound hTF. Whether the modulatory changes in the MCRC or TfR1 are geared to preserving PTEC cellular iron and/or as a means of maintaining systemic iron homeostasis remains to be determined. Interestingly, a study by Weiss *et al*. (31) reported decreased TfR1 and increased MCRC expression in kidney PT in response to iron overload, a treatment that can be considered the reciprocal of the iron chelation utilized in the current study. Furthermore, although data from humans detailing the proposed proximal tubule iron reabsorption mechanism are sparse, van Raaij *et al*. (11) have recently catalogued the distribution of DMT1 and FPN1 in human kidney and reported that the expression profiles match those reported in rodents, therefore signifying that the iron transport mechanism as characterized in rodents most probably exists in humans.

The pathophysiological implications of the findings detailed herein are of importance, especially relating to diseases featuring disruption of glomerular integrity, including diabetic nephropathy, membranous glomerulopathy, and focal segmental glomerular sclerosis. As a consequence of degeneration of the glomerular filtration barrier, more protein is filtered, resulting in more hTf entering the primary filtrate. Thus, the PT is presented with more iron to reabsorb, which in at least a third of humans results in PT iron deposition (11). Interestingly, in the streptozotocin-induced diabetic rat, there is a 4-fold increase in urinary iron excretion (32), an increase in renal TfR1 expression, and decreased DMT1 expression (33). Clearly, when diseased, the kidney responds to the altered filtrate that it is confronted with, and in the future it will be beneficial to understand and possibly manipulate this response to preserve the life of failing kidneys.

In conclusion, this report demonstrates that TfR1 is expressed apically in the epithelial cells of the PT and provides an inherent avenue for transport of hTf across the apical membrane. hTf uptake is mediated by the MCRC and TfR1, and both pathways are modulated at the level of the apical membrane in response to decreased cellular iron. The findings of this study have implications for the renal handling of iron, the homeostatic control of iron balance, and pathologies involving glomerular degeneration.

## Experimental procedures

### Immunostaining for TfR1

All animal protocols were approved and performed under a license issued for the use of experimental animals by the Danish Ministry of Justice (Dyreforsøgstilsynet), and methods were performed in accordance with local guidelines and regulations. Male C57BL/6J mice of 8 weeks of age were anesthetized with isoflurane and fixed by retrograde perfusion through the abdominal aorta with 4% paraformaldehyde (PFA) in PBS, pH 7.4. The tissue was dehydrated and embedded in paraffin by standard methods. Immunohistochemistry was performed on 2-μm-thick kidney sections as described previously (34) using a 1:2500-fold dilution of a mouse mAb against the TfR1 (H68.4, catalog number 13-6800, Thermo Fisher). Labeling was visualized by use of a peroxidase-conjugated secondary antibody for light microscopy (P448, Dako, Glostrup, Denmark) and visualized with 0.05% 3,3′-diaminobenzidine tetrachloride (Kemen Tek, Copenhagen, Denmark). Light microscopy was performed with a Leica DMRE microscope (35). The procedure for immunofluorescent labeling has been described in detail previously (36). TfR antibody was used at 1:1000 dilution. Primary antibody alone or secondary antibody alone controls were negative for immunolabeling. A Leica TCS SL confocal microscope with an HCX PL APO 63× oil objective lens (numerical aperture, 1.40) was used for imaging of labeled sections (Leica Microsystems, Ballerup, Denmark).

### Cell culture of rat PT WKPT and human PT HPCT cells

Immortalized cells originating from the S1 segment of WKPT (WKPT-0293 Cl.2) (30) or S1 segment from normal adult HPCT (29), kind gifts from Professor Ulrich Hopfer, Case Western Reserve University, were grown in a 1:1 mixture of Dulbecco's modified Eagle's medium (DMEM) and Ham's F-12 nutrient mixture with 10% (WKPT) or 5% (HPCT) fetal bovine serum, 100 units/ml penicillin, 100 μg/ml streptomycin, 5 μg/ml insulin, 4 μg/ml dexamethasone, 0.01 μg/ml epidermal growth factor, 1.2 mg/ml NaHCO_3_, and 5 μg/ml human apotransferrin (T2252, Sigma-Aldrich) added at 37 °C and 5% CO_2_. On reaching confluence, cells were passaged (WKPT passage number, <40; HPCT passage number, <100) twice a week.

### Modulation of cellular iron levels

To reduce cellular iron concentration, DFO (100 μm) was added to culture medium and cells incubated for 24h. This treatment significantly reduces cellular iron (9).

### Polarized transferrin flux experiments

To measure apical or basolateral uptake of Tf, WKPT cells were grown on Transwell permeable supports (0.4-μm pores and 1.12-cm^2^ surface area, Costar, Transwell-clear, Corning), and transepithelial electrical resistance (TEER) was measured using an EVOM meter (World Precision Instruments). Uptakes were only performed on monolayers with effective TEER values of >100 ohms × cm^2^ (10). WKPT TEER was not affected by DFO treatment (data not shown). Prior to assessing uptake, cells were incubated for 1 h in serum-free medium (±DFO), then washed three times with serum-free DMEM, and incubated with 0.32 μmol/liter A488-hTf (Molecular Probes) for 10 min. After washing three times in ice-cold PBS, cells were trypsinized, pelleted (1,000 × *g* for 5 min at 4 °C), and fixed in 4% PFA in PBS for 30 min at room temperature. Cells were washed in PBS and centrifuged at 1,000 × *g* for 5 min at 4 °C. This treatment was repeated twice, and then cells were stored at 4 °C in the dark prior to FACS analysis.

### FACS analysis

Cellular fluorescence was measured using a Beckman Coulter CyAn ADP cell sorter running Summit software (version 4.3). A 488 nm laser was used for excitation, and fluorescent signal was detected as a 530/30 nm band pass. The fluorescence of 10,000 events was measured from each sample replicate, and a mean value was obtained for three replicates per treatment group. To enable gating parameters to be set, control cells were also analyzed in the absence of Bodipy fatty acid (Fig. S1).

### Apical uptake experiments

WKPT cells were grown on coverslips in 24-well plates for 4 days in DMEM with 10% fetal bovine serum, 1% penicillin and streptomycin, 5 μg/ml insulin, 4 μg/ml dexamethasone, 0.01 μg/ml epidermal growth factor, and 5 μg/ml apotransferrin at 37 °C and 5% CO_2_ and then incubated in this medium plus or minus 100 μm DFO for 24 h. Cells were washed three times in serum-free DMEM at 37 °C and incubated for 1 h in serum-free DMEM (±DFO). Cells were washed three times with serum-free DMEM and incubated in serum-free DMEM containing either 0.32 μmol/liter Cy3-RAP or 0.32 μmol/liter 488-hTf with or without 16 μmol/liter unlabeled competitor ligand (unlabeled RAP or hTf) for 15 min at 37 °C. Cells were subsequently washed three times in ice-cold PBS, incubated for 5 min in serum-free DMEM at 37 °C, then washed in ice-cold PBS, and fixed in 4% PFA in PBS for 30 min at room temperature. Thereafter, cells were washed three times with 1× PBS and quenched with 0.1 m glycine in PBS for 5 min at room temperature. Cell nuclei were stained using Hoechst 33342 (Sigma), and coverslips containing adhered cells were mounted on glass sides with mounting medium (Dako).

### Image analysis and measurement of cellular fluorescence

Fluorescence images were captured on a Nikon C1 confocal microscope. The confocal settings were: pinhole, 100 μm; scan speed, 400 Hz unidirectional; format, 512 × 512. Optical section thickness was calculated at 0.8 μm. 3D optical stacks were collected for 4′,6-diamidino-2-phenylindole (DAPI) (405 mm), FITC (488 mm), and Texas Red (543 mm) using Nikon Image software. Image analysis was performed using ImageJ (37). To measure total cellular fluorescence, nonsaturated 3D image stacks were flattened to create a composite image, which was then split into green and blue channels. Mean background was calculated for each channel by selecting three areas with no visible fluorescence. Measurement of fluorescence in the green or blue channel was then performed using the Measure function in ImageJ. The corrected total region of interest fluorescence (CTRF; green channel) was calculated using Equation 1.

(Eq. 1) CTRF=Integrated density−Area×Mean background

CTRF was then normalized to the corrected total blue channel fluorescence. This analysis was performed on replicates of 5 or 6 cover slips.

### qPCR

Quantitative real-time PCR was used to quantify mRNA encoding the transcripts of interest essentially as described previously (38) with the exception that Takyon SYBR Green MasterMix (Eurogentec) was utilized for qPCRs. Primer sequences and the expected size (bp) of the amplicons are detailed in Table S1; primers were used at concentrations of 300–1200 nm. Three reference genes were tested, and the most stable genes (*B2m* and *Gapdh* for WKPT and *B2M* and *TUBB2A* for HPCT) were used for data correction.

### Immunofluorescence staining and quantification of TfR1 and megalin

WKPT or HPCT cells were plated onto glass coverslips, grown to confluence, and treated with 100 μm DFO for 24 h in standard culture medium. For megalin labeling, cells were fixed in ice-cold methanol:acetone (1:1) for 5 min on ice and allowed to dry before proceeding. For TfR1 staining, cells were fixed in 4% PFA in PBS for 30 min at room temperature. After washing three times in PBS, cells were incubated in blocking buffer (1% bovine serum albumin (BSA) in PBS) for 40 min at room temperature and incubated with primary antibodies overnight at 4 °C in a humidified chamber. Mouse anti-rat TfR1 antibody conjugated to R-phycoerythrin (PE) (1:200; BD Pharmingen) for WKPT, mouse monoclonal TfR1 antibody (H68.4; 1:200) for HPCT, mouse monoclonal anti-megalin antibody (clone G-9; 1:125; Santa Cruz Biotechnology) for WKPT, and rabbit polyclonal anti-megalin antibody (Bioss; 1:100) for HPCT were diluted in blocking buffer. Negative controls were incubated either in blocking buffer alone or with isotype control PE-conjugated nonspecific mouse IgG. Secondary anti-IgG antibody conjugated to Alexa Fluor 488 (Jackson ImmunoResearch Laboratories) was diluted 1:500 in PBS and incubated for 45 min at room temperature. Nuclei were counterstained with 0.8 μg/ml Hoechst-33342 in PBS for 5 min. After washing with water, coverslips were mounted with fluorescence mounting medium (Dako).

Fluorescence labeling was viewed on an imaging system with the following excitation/emission wavelengths (λex/em) for PE (λex/em 545/610 nm), Alexa 488 (λex/em 480/535 nm), and Hoechst (λex/em 360/460 nm) using a SOLA SM II white light LED engine (Lumencor) as light source, which was connected to a Zeiss Axiovert 200 M microscope (Carl Zeiss) equipped with a Fluar 40×, 1.3 numerical aperture oil immersion objective. Images were captured using a digital CoolSPAN ES CCD camera (Roper Scientific) using VisiView software (Visitron Systems GmbH). Background subtraction, intensity scaling, and analyses of images were performed using ImageJ/Fiji (39). For threshold area analysis, inclusive threshold cutoff was determined to include areas of interest, that is maximum fluorescent intensity, and exclude background and low intensity areas. The same threshold cutoff was applied to all images in a single experiment. A region approximately half the total area was used to select areas of similar cell densities, and the percentage area above threshold was determined and corrected for cell number. Ten images per condition were analyzed.

### Surface biotinylation and immunoblotting of TfR1

Cell culture flasks were coated with 125 μg/ml rat tail collagen (Sigma-Aldrich) diluted in 100 mm CH_3_COOH for at least 2 h prior to washout with PBS and cell plating. WKPT cells (3 × 10^6^/75 cm^2^) were grown to confluence and treated with 0.1 mm DFO for 16 h in normal culture medium. Surface biotinylation on the adherent monolayer was performed using a Cell Surface Biotinylation kit (Pierce) according to the manufacturer's instructions. Homogenate samples were collected after the cell lysis step. Protein concentration was determined by the method of Lowry *et a*l. (24). Equal amounts of protein were separated by SDS-PAGE, transferred to polyvinylidene fluoride membranes, and immunoblotted using mouse anti-TfR1 (clone H68.4; 1:5000; Thermo Fisher), rabbit anti-Na^+^/K^+^-ATPase (1:1000; Cell Signaling Technology), rabbit anti-GAPDH (1:40,000; Cell Signaling Technology), and species-specific horseradish peroxidase–coupled secondary antibodies (Jackson ImmunoResearch Laboratories). Signals were visualized using Immobilon chemiluminescence substrate (Millipore) and blue X-ray films. Densitometry analysis was performed using ImageJ/Fiji. Protein loading was controlled by Coomassie blue gel staining.

### Data processing and statistics

The two-sample Kolmogorov–Smirnov (KS) test was used to determine whether fluorescence frequency distribution data were statistically significantly different. Raw FACS analysis data were normalized for total cell number and analyzed using a KS software package (http://www.physics.csbsju.edu/stats/KS-test.n.plot_form.html).^3^ Frequency distributions were deemed to be significantly different if *p* was ≤0.05. ANOVA was used to compare the means of experimental groups, Dunnett's post hoc test was used for group comparisons, and Student's unpaired *t* test was used for pairwise comparisons where significance was assumed at *p* ≤ 0.05.

## Author contributions

C. P. S. conceptualization; C. P. S., W.-K. L., and F. T. resources; C. P. S., W.-K. L., M. H., F. T., and R. A. F. data curation; C. P. S., W.-K. L., M. H., S. B. P., F. T., and R. A. F. formal analysis; C. P. S. and R. A. F. supervision; C. P. S. and F. T. funding acquisition; C. P. S. and M. H. validation; C. P. S., W.-K. L., M. H., S. B. P., and R. A. F. investigation; C. P. S. visualization; C. P. S., F. T., and R. A. F. methodology; C. P. S. and F. T. writing-original draft; C. P. S. project administration; C. P. S., W.-K. L., F. T., and R. A. F. writing-review and editing.

## Supplementary Material

Supporting Information

## Footnotes

3 Please note that the JBC is not responsible for the long-term archiving and maintenance of this site or any other third party-hosted site.

## Footnotes

2 The abbreviations used are:

PT: proximal tubule
Tf: transferrin
MCRC: megalin–cubilin receptor complex
TfR1: transferrin receptor 1
hTf: holotransferrin
RAP: receptor-associated protein
PTEC: proximal tubule epithelial cell
DMT1: divalent metal transporter 1
FPN1: ferroportin 1
WKPT: Wistar–Kyoto rat proximal tubule
HPCT: human proximal tubule
DFO: desferrioxamine
TEER: transepithelial electrical resistance
DMEM: Dulbecco's modified Eagle's medium
Alexa 488 or A488: Alexa Fluor 488
RT-qPCR: quantitative RT-PCR
qPCR: quantitative real-time PCR
Lcn2-R: lipocalin 2 receptor
PFA: paraformaldehyde
CTRF: corrected total region of interest fluorescence
PE: R-phycoerythrin
λex/em: excitation/emission wavelengths
KS: Kolmogorov–Smirnov
ANOVA: analysis of variance
GAPDH: glyceraldehyde-3-phosphate dehydrogenase.
