# Supplementary material for: Proximal tubule transferrin uptake is modulated by cellular iron and mediated by apical membrane megalin–cubilin complex and transferrin receptor 1
Source: J Biol Chem. 2019 Mar 4;294(17):7025–36. doi: 10.1074/jbc.RA118.006390 (PMC6497946; doi:10.1074/jbc.RA118.006390)
Supplement: Supporting Information [file supp_294_17_7025__index.html]

Proximal tubule transferrin uptake is modulated by cellular iron and mediated by apical membrane megalin–cubilin complex and transferrin receptor 1 — Proximal tubule transferrin uptake — Proximal tubule transferrin uptake is modulated by cellular iron and mediated by apical membrane megalin–cubilin complex and transferrin receptor 1 — Proximal tubule transferrin uptake — Supporting Information 

# Proximal tubule transferrin uptake is modulated by cellular iron and mediated by apical membrane megalin–cubilin complex and transferrin receptor 1

## Supporting Information

- Supporting Information (to be published online) - Supplementary figures for the publication
