# Supplementary material for: Proximal tubule transferrin uptake is modulated by cellular iron and mediated by apical membrane megalin–cubilin complex and transferrin receptor 1
Source: J Biol Chem. 2019 Mar 4;294(17):7025–36. doi: 10.1074/jbc.RA118.006390 (PMC6497946; doi:10.1074/jbc.RA118.006390)
Supplement: Supporting Information [file supp_RA118.006390_141504_2_supp_295327_pnwr5k.pdf]

Supplementary Figure 1. Smith et.al.

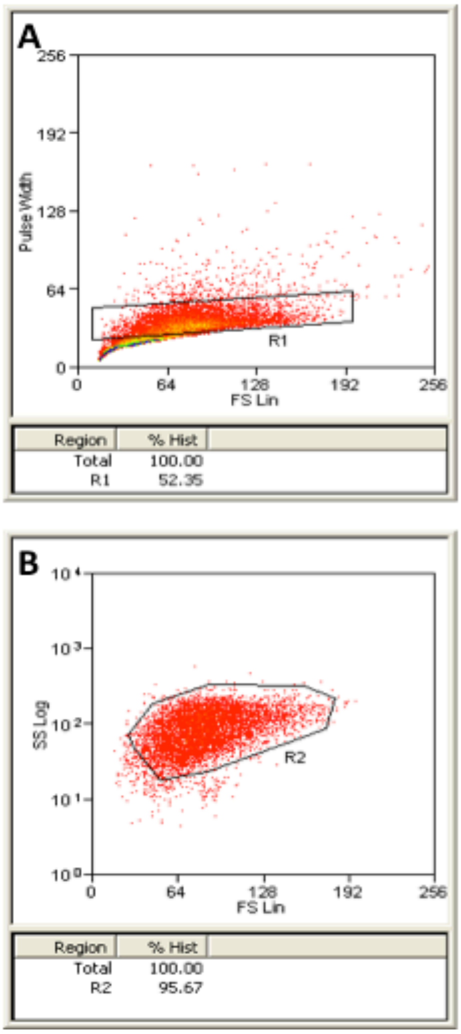

Supplementary Figure 2. Smith et.al.

A

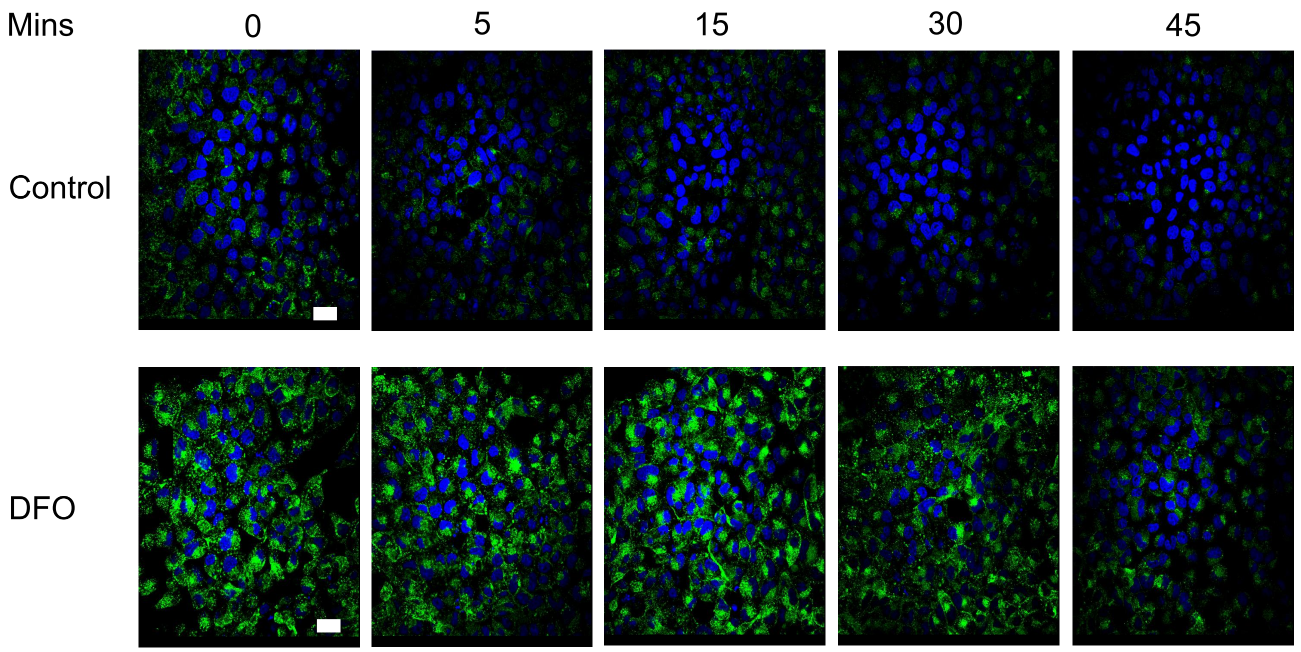

B

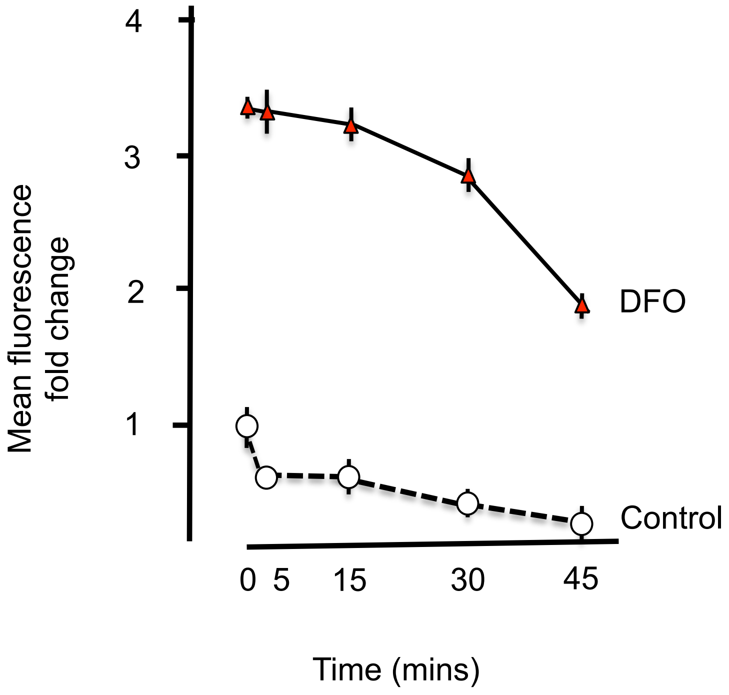

Supplementary Table 1. Smith et.al.

Primer	Sequence (5' – 3')	Genbank Accession No.	Product size (bp)	Reference
Rat Tfr1 forward	ATACGTTCCCCGTTGTTGAGG	NM_022712.1	112	Malik, IA et al. Cell Tissue Res 2011
Rat Tfr1 reverse	GGCGGAAACTGAGTATGGTTGA			
Rat Lrp2 forward	TGGAATCTCCCTTGATCCTG	NM_030827.1	161	Prabakaran, T et al. Nephrol Dial Transplant 2012
Rat Lrp2 reverse	TGTTGCTGCCATCAGTCTTC			
Rat Cubn forward	GCACTGGCAATGAACTAGCA	NM_053332.2	183	Prabakaran, T et al. Nephrol Dial Transplant 2012
Rat Cubn reverse	TGATCCAGGAGCACTCTGTG			
Rat Bact forward	AGATCAAGATCATTGCTCCTC	NM_031144.2	117	NCBI Primer Blast
Rat Bact reverse	ACTCATCGTACTCCTGCTTG			
Rat B2m forward	AGACCGATGTATATGCTTGC	NM_012512.2	109	NCBI Primer Blast
Rat B2m reverse	CAGATGATTCAGAGCTCCAT			
Rat Gapdh forward	AGGGCTCATGACCACAGT	NM_017008.4	112	NCBI Primer Blast
Rat Gapdh reverse	TGCAGGGATGATGTTCTG			
Human TFR1 forward	TGGCAGTTCAGAATGATGGA	NM_003234.3	86	Grisouard, J et al. Blood 2016
Human TFR1 reverse	AGGCTGAACCGGGTATATGA			
Human LRP2 forward	TGAAATTGGCTGCGCTGT	NM_004525.3	248	Jensen, LL et al. Physiol Rep 2014
Human LRP2 reverse	AGCTCCATCGGGGCAGTC			
Human CUBN forward	AATGGATGTGTGCAGCTCAG	NM_001081.3	153	Jensen, LL et al. Physiol Rep 2014
Human CUBN reverse	GGGGTTGCTCAAACACTCAT			
Human BACT forward	ACTGGGACGACATGGAGAAA	NM_001101.3	189	Thanasai J,et al,World Journal of Gastroenterology , 2010
Human BACT reverse	ATAGCACAGCCTGGATAGCA			
Human B2M forward	CTCCGTGGCCTTAGCTGTG	NM_004048.2	69	RTPrimerDB ID 3534
Human B2M reverse	TTTGGAGTACGCTGGATAGCCT			
Human TUBB2A forward	ATCAGCAAGATCCGGGAAGAG	NM_001069.3	82	Locati, M et al J Immunol 2002
Human TUBB2A reverse	CCGTGTCTGACACCTTGGGT			

Supplementary Figure 1. FACS gating. Representative plots showing forward scatter (x-axis) vs. pulse width (y-axis) for FACS events. Cell samples were gated according to their size and viability. Red dots represent analysed 'events'. A) Forward scatter reflected the cell-surface size. Events that did not have properties typical of cells or events of atypical cell size which represented debris or clumps of cells were excluded by R1 gating. B) R2 gating was based on size and cell granularity and represented the properties of single, viable cells.

Supplementary Figure 2. Pulse-chase time course of Alexa 488-hTf fluorescence in WKPT cells following 24hr DFO treatment. A) WKPT cells were incubated with Alexa 488-hTf for 30min (pulse) then switched to serum-free DMEM (chase) and images recorded thereafter at 0, 5, 15, 30 and 45min. Images were taken using a Nikon upright confocal microscope at a fixed exposure. At 0 min DFO treated cells had taken up more Alexa 488-hTf relative to control cells. Fluorescence decreased over time for both DFO treated and control cells, however cellular fluorescence in the DFO group remained significantly elevated compared to time matched controls. B) Mean fluorescence as a ratio of control group at time 0. DFO treated cells took up more Alexa 488-hTf than control cells and cellular Alexa 488-hTf fluorescence remained greater than control over the time course of the experimental period ($p<0.01$). Values represent mean ± SEM, n=3. Scale bar = 20 µm.

Supplementary Table 1 Primer sequences and predicted product sizes (bp) used for semi-quantitative PCR analysis.
